# Patient-level costs of staged unilateral *versus* immediate bilateral symmetrization mammoplasty in breast-conserving surgery

**DOI:** 10.1093/bjsopen/zrac073

**Published:** 2022-06-08

**Authors:** Yasmin Grant, Paul T. R. Thiruchelvam, Lana Kovacevic, Elias Mossialos, Ragheed Al-Mufti, Katy Hogben, Dimitri J. Hadjiminas, Daniel R. Leff

**Affiliations:** Department of BioSurgery and Surgical Technology, Imperial College London, London, UK; Breast Unit, Imperial College Healthcare NHS Trust, London, UK; Department of BioSurgery and Surgical Technology, Imperial College London, London, UK; Department of Health Policy, The London School of Economics and Political Science, London, UK; Breast Unit, Imperial College Healthcare NHS Trust, London, UK; Breast Unit, Imperial College Healthcare NHS Trust, London, UK; Breast Unit, Imperial College Healthcare NHS Trust, London, UK; Breast Unit, Imperial College Healthcare NHS Trust, London, UK; Department of BioSurgery and Surgical Technology, Imperial College London, London, UK; Breast Unit, Imperial College Healthcare NHS Trust, London, UK

## Abstract

**Background:**

Following therapeutic mammoplasty (TM), the contralateral breast may require a later balancing procedure to optimize shape and symmetry. The alternative is to offer patients simultaneous TM with immediate contralateral symmetrization via a dual-surgeon approach, with the goal of reducing costs and minimizing the number of subsequent hospital appointments in an era of COVID-19 surges. The aim of this cost–consequence analysis is to characterize the cost–benefit of immediate bilateral symmetrization dual-operator mammoplasty *versus* staged unilateral single operator for breast cancer surgery.

**Method:**

A prospective single-centre observational study was conducted at an academic teaching centre for breast cancer surgery in the UK. Pseudonymized data for clinicopathological variables and procedural care information, including the type of initial breast-conserving surgery and subsequent reoperation(s), were extracted from the electronic patient record. Financial data were retrieved using the Patient-Level Information and Costing Systems.

**Results:**

Between April 2014 and March 2020, 232 women received either immediate bilateral (*n* = 44), staged unilateral (*n* = 57) for breast cancer, or unilateral mammoplasty alone (*n* = 131). The median (interquartile range (i.q.r.)) additional cost of unilateral mammoplasty with staged *versus* immediate bilateral mammoplasty was €5500 (€4330 to €6570) per patient (*P* < 0.001), which represents a total supplementary financial burden of €313 462 to the study institution. There was no significant difference between groups in age, Charlson comorbidity index, operating minutes, time to adjuvant radiotherapy in months, or duration of hospital stay.

**Conclusion:**

Synchronous dual-surgeon immediate bilateral TM can deliver safe immediate symmetrization and is financially beneficial, without delay to receipt of adjuvant therapy, or additional postoperative morbidity.

## Teaser

Synchronous dual-surgeon immediate bilateral therapeutic mammoplasty can deliver safe immediate symmetrization and is financially beneficial, without delay to receipt of adjuvant therapy, or additional postoperative morbidity in breast conservation.

## Introduction

Breast cancer is the most common cancer affecting women in the USA and Western Europe^1,2^, with the majority of patients being treated by breast-conserving surgery (BCS)^3^, followed by adjuvant radiotherapy^4–10^. There are data to support the survival advantage of BCS compared with mastectomy, independently of measured confounders and it should be given priority in suitable candidates^11^. Therapeutic mammoplasty (TM) extends the boundaries of BCS by combining breast reduction and mastopexy techniques with tumour excision, preserving natural breast cosmesis and circumventing the need for mastectomy^12,13^. Following TM, the contralateral breast may require a later balancing procedure to optimize shape and symmetry^14^. This may be performed immediately or as a staged procedure, depending on several factors, including patient choice. The disadvantage of staged contralateral symmetrization mammoplasty is that in the aftermath of the index cancer surgery, the patient is left asymmetric with subsequent impact on quality of life, confidence, and self esteem^15^.

The alternative is to offer patients simultaneous TM with immediate contralateral symmetrization with the goal of improving aesthetic outcomes, enhancing quality of life, and minimizing the number of subsequent clinic visits, hospital appointments, and operative procedures. The latter has come sharply into focus because of the recent SARS-CoV-2 virus (COVID-19) pandemic giving evidence to suggest that patients with cancer are regarded as a vulnerable group^16,17^. In this context, immediate symmetrization surgery may reduce the risk of nosocomial COVID-19-related infections by curtailing healthcare contact points and has been recommended by the British Association of Plastic Reconstructive and Aesthetic Surgeons^18^. Moreover, a recent Canadian study of 48 patients who underwent immediate bilateral symmetrizing TM demonstrated high levels of patient-reported satisfaction and psychosocial outcomes with comparable oncological safety and complication rates^19^. This notwithstanding, immediate symmetrization is arguably more technically challenging given the need to predict radiotherapy-related shrinkage of the treated breast^20^. Theoretically, there is the potential for delay to adjuvant therapies if complications ensue because of more extensive surgery. Moreover, logistical factors including protracted operating times and theatre inefficiencies mean that it is challenging for a single operator to offer an immediate bilateral mammoplasty service.

One solution is to offer a synchronous two-consultant team approach with the goal of facilitating immediate symmetrization, reducing operating time^21,22^, maximizing list utilization^23^, and theoretically reducing costs^24^ associated with the second hospital episode in a staged mammoplasty approach. A dual-operator approach is postulated to confer better control of the operative field, intraoperative recognition of technical errors, and better assistance for the primary surgeon, rendering a technically complex operation more straightforward^25^, but to date the approach has not been extrapolated to BCS. With TM, a second consultant may improve preoperative and intraoperative decision-making with a readily available second opinion^26^. Moreover, there is evidence that a dual-surgeon approach enhances theatre productivity with efficient use of surgeon time^22^ and streamlines progress of the operating list^24^. A good practice guideline in oncoplastic surgery published in 2021^26^ recommended a two-team immediate symmetrization approach to shorten operating time and reduce complication rates in implant reconstruction^27^. Moreover, a study of 116 patients undergoing bilateral mastectomy by a co-surgeon team also demonstrated a reduction in overall surgical time^28^. Critically, this recommendation is without an evidence base for mammoplasty and furthermore, previous dual-team approaches have overlooked potential cost opportunities.

Although the financial burden of delayed reconstructive surgery and symmetrization has been hypothesized, there are no published studies that define cost differentials between immediate single-stage and delayed two-stage symmetrization mammoplasty in BCS. Potential excess spending associated with delayed second-stage symmetrization with an additional theatre episode may impose unnecessary expenditure to health institutions. Despite this, a dual-operator technique has not been described for TM and the benefits for patients undergoing BCS are unknown. The aim of this study was to derive patient-level information costs (PLICS), a standardized method of cost information, in a UK single-site institution to compare individual cost drivers between patients undergoing delayed *versus* immediate symmetrization.

## Methods

### Ethical approval/service board approval

This study was registered at Imperial College Healthcare NHS Trust (ICHNT), London, UK as a service evaluation (under service evaluation board identification number 309).

### Patient identification, inclusion, and exclusion criterion

Prospective contemporaneous operative records held on the electronic record (Cerner^®^) were used to identify and extract source data, including demographic, clinica,l and procedural information. Patients receiving TM either with immediate or delayed symmetrization surgery at ICHNT between 1 April 2014 and 31 March 2020 were identified from operative records. The patients underwent unilateral alone, immediate, or delayed symmetrization due to the nature of referrals to individual surgeons at the trust. The choice of unilateral mammoplasty with or without immediate/delayed symmetrization was performed using clinical judgement or at the patient’s request. Immediate bilateral symmetrization was not mandatory across the unit. For this study, procedures meeting inclusion criteria were reduction mastopexy techniques, including removal of the skin and breast parenchyma to treat invasive (ductal, lobular, mucinous, or papillary carcinoma) or preinvasive (ductal carcinoma *in situ* or lobular carcinoma *in situ*) breast cancer with BCS. Specifically, these techniques included wise pattern reduction^29^, Le Jour-type vertical scar^30^, modified Benelli^31^, lateral wedge^32^, crescent^33^, central^34^, racket^35^, and melon slice^36^.

### Data sources

Pseudonymized data for clinicopathological variables and procedural care information, including the type of initial BCS and subsequent reoperation(s), were extracted from the electronic patient record (Cerner^®^, 2021 Cerner Corporation, Kansas City, Kansas, USA). Financial data were retrieved for patients who underwent TM with or without immediate or delayed symmetrization mammoplasty over the study interval. PLICS (CostMaster, 2021 Civica, London, UK) is a software package used to collate patient-level data on financial outcomes prospectively and systematically^37^. The main unit of observation was the attending episode and the line-item costs for patient episodes were recorded, including medical consultation, nursing, pathology, radiology, operating theatre, and supplies costs.

### Financial outcome data

A cost–consequence analysis (CCA) was conducted, which is a form of economic evaluation for disaggregated costs^38^, using PLICS. A CCA involves a broad assessment of costs under the broadest perspective possible, which allows individual decision-makers to choose the combination of costs most relevant to their decision context according to their chosen perspective, which may be narrower than the CCA perspective^39^. Total costs relating to immediate bilateral and delayed unilateral symmetrization surgery were obtained for inpatient episodes only. Direct medical costs included consultant, ward stay, theatre, nursing and other health professionals’ time, use of pharmaceutical products, and diagnostic and interventional procedure costs, including consumables. Direct non-medical costs were captured as capital overhead costs. Duration of hospital stay (days) was recorded. Costs were expressed in Euro and rounded to the nearest integer. The exchange rate used was £1 to 1.19 Euro as of 3 April 2022. The primary outcome was the total cost differential of symmetrization surgery between immediate symmetrization, delayed symmetrization, and unilateral mammoplasty alone.

### Clinical outcome measures

Using the Cerner Electronic Health Record, the type of index mammoplasty was recorded and included wide local excision with or without radiological guidance, with or without sentinel lymph node biopsy (SLNB), with or without axillary lymph node dissection (ALND), and with or without immediate symmetrizing TM performed concurrently by dual consultant surgeons. Procedural data regarding second-stage contralateral mammoplasty performed by a single operator were collected and the time delay to symmetrization was recorded. Comorbidity was assessed using the Charlson comorbidity index (CCI), a validated weighted index that estimates mortality risk from co-morbid disease^40^. Clinical data unavailable on the patient electronic health record were excluded. Exploratory outcomes included demographic and clinical characteristics that predict the total cost of immediate *versus* delayed symmetrization surgery. Secondary outcomes included time to receipt of adjuvant radiotherapy, unplanned reoperation, or re-admission to hospital following discharge home with either local or systemic complications related to surgery within 30 days from procedure according to the Clavien–Dindo classification^41^.

### Statistical analysis

Normality tests were performed to estimate the appropriateness of parametric estimators, and inferential statistics were employed according to these assumptions. Associations between categorical variables were examined using chi-squared tests for linear trends. Associations between direct costs of index mammoplasty with immediate or delayed symmetrization were examined using Mann–Whitney *U* and Kruskal–Wallis tests. Differences between groups were deemed to be statistically significant at the 5 per cent level. Statistical analysis was conducted using Stata^®^ version 14.2 (StataCorp, College Station, Texas, USA). A multivariate linear regression was used to understand the association between total care costs and patient characteristics, including age, clinicopathological features, and receipt of adjuvant therapies.

## Results

### Patient demographics and procedural information

Between 1 April 2014 and 31 March 2020, 101 women received either immediate bilateral (*n* = 44) or staged unilateral (*n* = 57) symmetrization for breast cancer. A further 131 patients underwent unilateral mammoplasty only without any contralateral balancing procedure. There was no significant difference between groups in median (interquartile range (i.q.r.)) ages in years: 59 (17) years for immediate bilateral group; 60 (22) years for the staged unilateral group; and 58 (14) years for the mammoplasty only group (*P* = 0.879). As summarized in *Table 1*, there was no significant difference in CCI between cohorts.

**Table 1:** Demographics for unilateral-alone, unilateral staged, bilateral immediate mammoplasty groups

	Unilateral alone (*n* = 131)	Unilateral staged (*n* = 57)	Immediate bilateral (*n* = 44)	χ^2^	*P*
**Age (years)**					
20–30	1 (0.8)	0 (0)	0 (0)	6.670	0.879
31–40	4 (3.1)	1 (2)	3 (7)		
41–50	26 (19.8)	13 (23)	5 (11)		
51–60	41 (31.3)	16 (28)	17 (39)		
61–70	32 (24.4)	12 (21)	10 (23)		
70–80	23 (17.6)	12 (21)	7 (16)		
80–90	4 (3.1)	3 (5)	2 (5)		
**Charlson comorbidity index**					
2	5 (3.8)	1 (2)	3 (7)	8.347	0.595
3	26 (19.8)	14 (25)	5 (11)		
4	39 (29.8)	17 (30)	17 (39)		
5	29 (22.1)	9 (16)	7 (16)		
6	27 (20.6)	14 (25)	12 (27)		
7	5 (3.8)	2 (4)	0 (0)		

Values are *n* (%) unless otherwise indicated.

There was no significant difference between T category (*P* = 0.463), nodal status (*P* = 0.130), or type of disease (invasive/non-invasive/mixed) (*P* = 0.726).

Of the 44 patients undergoing immediate bilateral symmetrization, 33 (75 per cent) included an axillary procedure, of which 25 patients (57 per cent) received an SLNB and eight patients (18 per cent) received an ALND.

Of the 57 patients who had staged unilateral symmetrization, 30 patients underwent an axillary procedure (53 per cent), which included 21 with SLNB (37 per cent) and nine with ALND (16 per cent). The median (i.q.r.) time to second-stage contralateral symmetrization was 14 (9–19) months.

### Clinical outcomes

*Table 2* details procedural characteristics and complications.

**Table 2:** Procedural characteristics and receipt of adjuvant therapy for unilateral-alone, unilateral staged, bilateral immediate mammoplasty groups

	Unilateral alone (*n* = 131)	Unilateral staged (*n* = 57)	Bilateral immediate (*n* = 44)	χ^2^	*P*
**Procedural characteristics Type of mammaplasty**					
Wise	39 (29.8)	12 (23)	21 (48)	NA	NA
Lejour	24 (18.3)	3 (6)	18 (41)		
Round block	33 (25.2)	1 (2)	5 (11)		
Central	1 (0.8)	1 (2)	0 (0)		
Melon slice	0 (0)	1 (2)	0 (0)		
Racket	2 (1.5)	0 (0)	0 (0)		
Lateral radial	0 (0)	1 (1)	0 (0)		
Wedge	1 (0.8)	0 (0)	0 (0)		
Excluded	28 (21.4)	37 (65)	0 (0)		
**Operating time**					
Minutes	86 (61–102)	138 (125–147)	113 (92–164)	214.40	0.202
**Re-excision of margins with/ without mastectomy**					
Yes	26 (19.8)	12 (23)	8 (18)		
No	103 (78.6)	41 (77)	36 (82)	0.302	0.351
Unknown	2 (1.5)	4 (7)	0 (0)		
**Postoperative complication (Clavien–Dindo classification)**					
None	121 (92.3)	45 (92)	43 (98)		
1	0 (0)	0 (0)	0 (0)		
2	3 (2.3)	2 (4)	0 (0)	1.567	0.457
3	4 (3.1)	2 (4)	0 (0)		
4	0 (0)	0 (0)	0 (0)		
5	0 (0)	0 (0)	0 (0)		
Unknown	0 (0)	0 (0)	0 (0)		
**Receipt of adjuvant therapy**					
**Radiotherapy**					
Yes	86 (65.6)	41 (71)	34 (77)	4.693	
No	33 (25.2)	6 (11)	8 (18)		0.096
Unknown	12 (9.2)	10 (18)	2 (5)		
**Chemotherapy**					
Yes	35 (26.7)	15 (26)	34 (77)	2.874	
No	88 (67.2)	32 (56)	9 (20)		0.579
Unknown	8 (6.1)	10 (18)	1 (2)		
**Endocrine therapy**					
Yes	81 (61.8)	41 (72)	31 (70)		
No	19 (14.5)	6 (11)	8 (18)	0.087	0.957
Unknown	31 (23.7)	10 (18)	5 (11)		
**Neoadjuvant therapy**					
Yes	11 (8.4)	7 (12)	5 (11)		
No	112 (85.5)	41 (72)	39 (89)	1.175	0.556
Unknown	8 (6.1)	9 (16)	0 (0)		
**Herceptin**					
Yes	11 (8.4)	4 (7)	4 (9)		
No	91 (69.5)	39 (68)	37 (84)	0.085	0.959
Unknown	29 (22.1)	14 (25)	3 (7)		

Values are *n* (%) unless otherwise indicated. Values are median (i.q.r) for operating time. NA, not applicable.

#### Operating time

Median (i.q.r.) theatre time in minutes of unilateral mammoplasty alone was 86 (61–102) min, 138 (125–147) min for unilateral staged mammoplasty, and 113 (92–164) min for immediate bilateral mammoplasty. There was no significant difference in operating time between unilateral staged and bilateral immediate symmetrization mammoplasty (*P* = 0.202).

#### Tumour biology

As summarized in *Table 3*, there was no significant between-group difference in tumour biology, including grade (*P* = 0.303), size (*P* = 0.916), lymph node status (*P* = 0.130), oestrogen receptor positivity (*P* = 0.957), progesterone receptor positivity (*P* = 0.278), and HER2 receptor positivity (*P* = 0.959).

**Table 3:** Tumour biology for unilateral-alone, unilateral staged, bilateral immediate mammoplasty groups

	Unilateral alone (*n* = 131)	Unilateral staged (*n* = 57)	Bilateral immediate (*n* = 44)	χ^2^	*P*
**High grade (G3)**					
Yes	34 (19.8)	18 (32)	9 (20)		
No	84 (64.1)	31 (54)	32 (72)	2.388	0.303
Unknown	13 (9.9)	16 (28)	3 (7)		
**Size (mm)**					
0–10	18 (13.7)	11 (19)	7 (16)		
11–20	63 (48.1)	15 (26)	12 (27)		
21–30	52 (39.7)	9 (16)	11 (25)		
31–40	23 (17.6)	6 (11)	4 (9)	4.606	0.916
41–50	11 (8.4)	2 (4)	3 (7)		
50+	21 (16.0)	7(12)	2 (5)		
Unknown	14 (10.7)	7 (12)	5 (11)		
**Nodal metastasis**					
Yes	27 (20.6)	15 (46)	6 (14)		
No	68 (51.9)	26 (46)	31 (70)	4.073	0.130
Unknown	36 (27.5)	16 (28)	7 (16)		
**Oestrogen receptor positivity**					
Yes	81 (61.8)	34 (60)	31 (70)		
No	19 (14.5)	9 (16)	8 (18)	0.087	0.957
Unknown	31 (23.7)	18 (25)	20 (45)		
**Progesterone receptor positivity**					
Yes	67 (51.1)	25 (44)	28 (64)		
No	32 (24.4)	18 (32)	12 (27)	5.094	0.278
Unknown	32 (24.4)	14 (25)	17 (39)		
**Human epidermal growth factor 2 receptor positivity**					
Yes	11 (8.4)	4 (7)	4 (9)		
No	91 (69.5)	39 (68)	37 (84)	0.085	0.959
Unknown	29 (22.1)	14 (25)	3 (7)		

Values are *n* (%) unless otherwise indicated.

#### Rates of re-excision for positive margins

Subsequent reoperation for positive margins occurred in six (14 per cent) of 44 patients undergoing immediate bilateral symmetrization, 12 (23 per cent of patients undergoing unilateral staged symmetrization, and 26 (20 per cent) of patients undergoing unilateral-alone mammoplasty (*P* = 0.351). All re-operative procedures (*n* = 44) were performed for close/positive margins defined as 0–1 mm from ink as per Association of Breast Surgery guidelines^42^.

#### Re-operative procedures

Of the bilateral immediate symmetrization cohort with positive margins, one patient underwent completion mastectomy without immediate reconstruction, one patient required completion mastectomy with deep inferior epigastric perforators (DIEP) flap, and four patients proceeded to unilateral re-excision for positive margins. Of the unilateral staged symmetrization cohort with positive margins, nine patients had completion mastectomy without immediate reconstruction and three patients required unilateral re-excision for positive margins. Of the unilateral-alone cohort with positive margins, 15 patients required completion mastectomy without immediate reconstruction, one patient had completion mastectomy with DIEP flap, and nine patients had unilateral re-excision for positive margins.

#### Postoperative complications

There was no statistically significant difference in the rate of complications by procedural cohort (*Table 2*). Postoperative complication at 30 days occurred in none of the 44 patients undergoing immediate bilateral symmetrization and four (8 per cent) of the 57 patients receiving staged symmetrization. In the staged mammoplasty group, two (4 per cent) patients received antibiotics for postoperative infection (grade II) and two patients (4 per cent) returned to theatre for washout of haematoma on index admission (grade IIIb). Of the 131 patients undergoing unilateral-alone mammoplasty, three patients (3 per cent) received antibiotics for postoperative infection (grade II) and four patients (3 per cent) returned to theatre for postoperative infection and washout (grade IIIb). Unplanned return to theatre episodes all occurred in the index admission. There were no delays in wound healing or skin necrosis.

#### Duration of hospital stay

The operating approach had no significant impact on duration of hospital stay (*P* = 0.134). Median (i.q.r.) length of stay for patients was 0 (0–1) days for patients receiving bilateral immediate symmetrization mammoplasty (*n* = 44), 0 (0–1) days for patients receiving staged unilateral mammoplasty (*n* = 57), and 0 (0–1) days for patients receiving unilateral mammoplasty alone (*n* = 131).

#### Adjuvant therapy

Regardless of procedural category, there was no significant difference in the time to radiotherapy (*P* = 0.770) or chemotherapy (*P* = 0.671). Median (i.q.r.) time to adjuvant radiotherapy was two (1–2) months for patients receiving bilateral immediate mammoplasty (*n* = 44), three (1–8) months for patients receiving unilateral staged mammoplasty (*n* = 36), and three (0–9) months for patients receiving unilateral-alone mammoplasty (*n* = 92). Median (i.q.r.) time to adjuvant chemotherapy was four (3–5) months for patients receiving bilateral immediate mammoplasty (*n* = 2), four (1–6) months for patients receiving unilateral staged mammoplasty (*n* = 10), and four (2-6) months for patients receiving unilateral-alone mammoplasty (*n* = 14).

#### Time to symmetrization

Regarding patients receiving unilateral staged mammoplasty (*n* = 34), the median (i.q.r.) time to symmetrization was 14 (10) months.

#### Hospital attendances

There was a statistically significant between-group difference in the number of hospital attendances favouring immediate bilateral symmetrization (*P* = < 0.001). The median (i.q.r.) number of hospital attendances including outpatient clinic, preoperative assessment, and theatre episodes was 8 visits (6) in the bilateral immediate symmetrization (*n* = 44), 16 visits (10) in the unilateral staged symmetrization (*n* = 46), and 14 visits (7) in the unilateral-alone cohort (*n* = 122).

### Financial outcomes

**Fig. 1:**
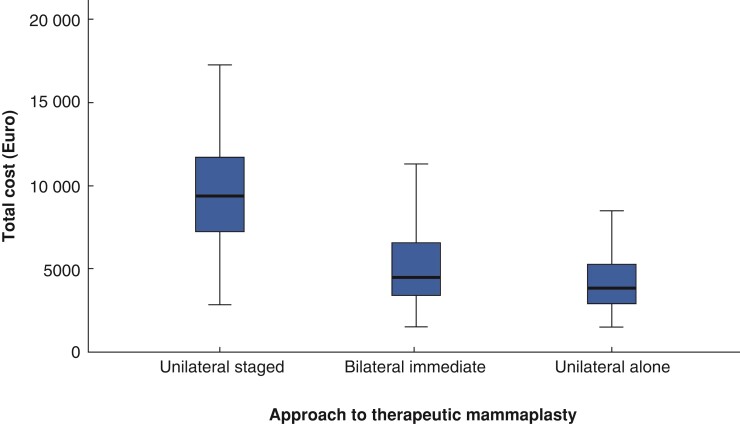
Total median patient-level costs of therapeutic mammoplasty (Euro)

Patient-level costs according to procedural cohort are summarized in *Table S1* and illustrated in *Fig. 1*. The additional median cost (i.q.r.) of unilateral staged mammoplasty is €5500 (€4330 to €6570) per patient, which is 1.5 times more costly compared with bilateral immediate mammoplasty and found to be statistically significant (*P* < 0.001). The overall cost of unilateral staged mammoplasty (*n* = 57), including index procedure with subsequent symmetrization, was €535 106 with a median (i.q.r.) cost of €9368 (€6982–11 646). The median (i.q.r.) cost of the index mammoplasty procedure was €4118 (€2521–5619) and median (i.q.r.) cost of subsequent symmetrization was €4327 (€2877–5856). The overall total cost of bilateral immediate mammoplasty (*n* = 44) was €258 020 with a median (i.q.r.) cost per patient of €4696 (€2724–6745). The median cost (i.q.r.) of unilateral-alone mammoplasty was €3868 (€2333–4758). Extrapolation of subsequent contralateral symmetrization at the median cost difference for unilateral patients yet to receive symmetrization (*n* = 131) represents a substantial additional cost of €566 783 over a 5-year interval.

### Causal estimates relating to cost drivers

The results of multivariate linear regression (*Table S2*) suggest that the most important, and indeed the only statistically significant, predictor of total care costs was the type of symmetrization, with bilateral immediate symmetrization resulting in significantly lower costs when compared with unilateral staged symmetrization (coefficient €3440.84, *P* < 0.001). Subsequently, a probit regression model was estimated, regressing the treatment variable on age, clinicopathological features, and receipt of adjuvant therapies. The results of the model (*Table S3*) indicate that none of the covariates of interest was a statistically significant predictor of treatment.

## Discussion

In this cost–consequence analysis, delayed symmetrization in BCS resulted in an additional cost of €313 462 when compared with immediate bilateral mammoplasty. Furthermore, it was demonstrated that immediate bilateral surgery did not lead to significant delays to adjuvant radiotherapy nor increase perioperative complication rates. This economic assessment using granular and comprehensive data has demonstrated a potential saving for healthcare providers of oncoplastic surgery. Healthcare spending on cancer in the UK is rising annually, and healthcare providers are seeking opportunities to reduce the cost burden and optimize cost^43,44^. Economic evaluations are thus being utilized increasingly to inform and improve healthcare quality across worldwide populations^45^.

Patient-level costing represents a robust analysis of individual patient charges of symmetrization surgery for breast cancer for the entirety of the hospital admission in the UK^46^. The data set utilizes a standardized method that helps to identify the relationship between patient characteristics and cost and assists the healthcare institution in maximizing resource allocation to improve efficiencies and support benchmarking. By mapping the steps of a patient’s care, the cost of each step can be calculated directly and are likely to be more accurate and actionable^47^. Specifically, it reflects the causality of costs in the BCS and other clinical pathway, tracing which type of activity is incurring cost for each patient in full granularity, which could be generalizable to healthcare provision in the UK. To date, there has been a paucity of high-quality evidence to support the benefits of immediate bilateral symmetrization in breast cancer surgery; however, this study demonstrates a clear and significant financial benefit at the hospital provider level.

Applying the current institutional cost estimates to breast cancer institutions in the UK could provide compelling evidence to minimize second-stage operations in oncoplastic surgery, thus reducing cost burden. Between 1 November 2016 and 31 October 2017, 685 TMs were identified to have been performed in 198 surgical units in England in a Getting It Right First Time (GIRFT) study^48^. Using the GIRFT procedural figure for TM could represent a potential additional national extrapolated cost of €2 963 712 for staged symmetrization per annum. A further example in a study of California Hospital financial statements identified mean costs of $36–37 per minute in the operating room, with minimal variation by setting or institutional characteristic^49^. Almost half of these costs are ‘indirect’ (expenses generated by non-revenue centres), including security and parking, which are outside of the control of the clinicians or departments^50^. The current findings suggest that overall cost could be modified with consideration of a dual-consultant immediate bilateral symmetrization approach as recommended by recent good practice guidelines^25^, as saving a second-stage procedure may realize substantial operating rooms savings for institutions in the UK.

Importantly, dual-surgeon immediate symmetrization was not associated with significant unplanned return to theatres or readmissions. There was no significant difference in positive margins or reoperation rate in the immediate bilateral cohort, echoing previous studies that have demonstrated that level II oncoplastic surgery results in low positive-margin rates^50^.

Furthermore, there was no significant difference in operating time between unilateral-alone and bilateral symmetrization, suggesting that there was no additional anaesthetic exposure. Interestingly, commensurate with the current findings, a previous study of 116 patients undergoing bilateral breast surgery by a co-surgical team also demonstrated a reduction in overall surgical time^28^. Taken together, these data suggest that up-front balancing mammoplasty procedures are safe and can be performed without undue increase in complication rates, or overall theatre time.

While more speculative, the immediate dual-operator approach may improve health-related quality of life for women who would traditionally experience a protracted duration of time with poor cosmetic outcomes following BCS. The median waiting time to subsequent symmetrization was 14 months in the study institution, which is a considerable amount of time for many women experiencing the psychological morbidity of asymmetry. Surgical waiting lists are usually explained by a ‘lack of resources’ or, more specifically, a ‘lack of surgeons’^23^; however, it is hoped that the approach described here demonstrates how a radical review of theatre practices and surgeon collaboration may optimize operating theatre time, improve surgical efficiency, and significantly reduce the number of patients on waiting lists for symmetrization surgery. The latter is critical in the era of the COVID-19 pandemic when providers are desperately seeking to streamline resources, optimize patient care, and reduce contact points or number of treatments in the hope of reducing transmission. This relates to data suggesting that hospital treatments increase the likelihood of transmission of disease and surgery seems to increase the risk of a severe course, including risk of intensive care admission^16^, mechanical ventilation^17^, and death^16,51^. Single-stage immediate symmetrization mammoplasty is therefore considered to be preferable, eliminating the need for multiple surgical procedures over extended timeframes, while enhancing patient safety by reducing the potential for hospital-related disease transmission.

The approach described herein could be further extended to other hospitals in the UK if the funds of a delayed symmetrization procedure were redirected to appoint an extra surgeon for attendance on oncoplastic lists; however, this analysis has demonstrated that a transition to immediate bilateral balancing mammoplasty at 50 per cent of activity levels can be achieved without the need for additional surgeon resource. Improving the complex system of theatre efficiency with dual-surgeon presence, without the resultant need for extra theatre time, or hospital bed availability could therefore achieve the synchronous aims of reducing waiting times, while significantly reducing the cost burden to hospital providers.

The drawbacks of this study include the relatively small, heterogeneous sample size with exclusions due to inconsistency of availability of patient-level costs in the PLIC software due to coding emissions and/or errors, which in turn limits the validity of the regression analysis. Furthermore, clinicopathological details, including smoking status, BMI, weight of mammoplasty specimen, and time to adjuvant chemotherapy were not available. In addition, patient and aesthetic outcomes were not collected prospectively. This study provides a cross-sectional estimate of differences in costs and did not rely on a decision analytic model. A further limitation of this study is the inability to ascertain the added cost of a second consultant surgeon’s remuneration fees to facilitate dual operating and the feasibility of a two-surgeon approach in smaller hospitals.

There are persuasive motivations to promote the facilitation of co-surgeon operating to provide patients with the option of immediate bilateral symmetrization in the COVID-19 pandemic era and beyond. The refined CCA economic approach has identified relevant costs and outcomes, providing a broader and richer source of economic information increasingly needed by healthcare decision-makers. This study demonstrates the magnitude of added patient-level costs of delayed symmetrization after BCS, generates a hypothesis, and establishes a framework for further definitive patient-level cost–consequence analyses.

## Supplementary Material

zrac073_Supplementary_Data

## Data Availability

The authors confirm that the data supporting the findings of this study are available within the article and its Supplementary Material. Raw data that support the findings are available from the corresponding author on reasonable request.
