# Supplementary material for: Patient-level costs of staged unilateral versus immediate bilateral symmetrization mammoplasty in breast-conserving surgery
Source: BJS Open. 2022 Jun 8;6(3):zrac073. doi: 10.1093/bjsopen/zrac073 (PMC9176201; doi:10.1093/bjsopen/zrac073)
Supplement: zrac073_Supplementary_Data [file zrac073_supplementary_data.docx]

| **Table S1. Patient-level costs and theatre time of unilateral alone, unilateral staged and bilateral immediate mammoplasty** | | | | |
| --- | --- | --- | --- | --- |
|  | **Cost (£)** | | |  |
|  | **Unilateral alone (n=131)** | **Unilateral staged (n=57)** | **Bilateral immediate (n=44)** | **p-value** |
| Total | 3,259 (1,243 – 15,595) | 7,892 (3,930 – 14,502) | 3,957 (1,302-15,807) | <0.001 |
| Operating room | 432 (25-4137) | 720 (44-6618) | 1149 (125-8125) | <0.001 |
| Pathology | 177 (0-2680) | 258 (4-4261) | 449 (0-1253) | <0.001 |
| Radiology | 42 (0-476) | 412 (9 – 1331) | 115 (0-1170) | <0.001 |
| Consultant | 442 (24-1609) | 1587 (533-5330) | 481 (106-4386) | <0.001 |
| **Cost consequence analysis: Patient-level costs of unilateral staged versus bilateral immediate mammaplasty (£)** | | | | |
|  | **Unilateral alone (n=109)** | **Unilateral staged (n=57)** | **Bilateral immediate (n=44)** | **Difference** |
| Total | **n/a** | 8,241 (2,452-15,144) | 4,132 (1,359 -16,600) | 4,108 (1,093 to 13,785) |
| Values are median (range)   \| **Table S2. Multivariate linear regression of total costs on treatment, patient characteristics and therapies** \| \| \| \| \| \| \| \| \| \| --- \| --- \| --- \| --- \| --- \| --- \| --- \| --- \| --- \| \| **Total Cost** \| \| **Coef** \| **Std. Err. (robust)** \| **t** \| **P>\|t\|** \| \| **95% CI** \| \| \| **Treatment (*bilateral immediate*)** \| \| -2898.79 \| 731.07 \| -3.97 \| .000 \| \| -4363.29 \| -1434.28 \| \| **Age** \| \| -35.44 \| 32.73 \| -1.08 \| .284 \| \| -101.00 \| 30.13 \| \| **Lymph node metastasis** \| \| -705.55 \| 858.33 \| -0.82 \| .415 \| \| -2424.99 \| 1013.88 \| \| **Radiotherapy** \| \| 845.34 \| 1511.76 \| 0.56 \| .578 \| \| -2183.08 \| 3873.75 \| \| **ER positivity** \| \| -334.21 \| 665.60 \| -0.50 \| .618 \| \| -1667.56 \| 999.15 \| \| **Size** \| \| 226.56 \| 334.04 \| 0.68 \| .500 \| \| -442.61 \| 895.73 \| \| **Constant** \| \| 9555.23 \| 2630.68 \| 3.63 \| .001 \| \| 4285.35 \| 14825.10 \| \|  \|  \| \|  \|  \|  \| No. of obs = 63  F(6, 56) = 3.34  Prob > F = 0.007  R-squared = 0.2355  Root MSE = 2852.4 \| \| \| \|  \|  \| \|  \|  \|  \| \|  \|  \| \|  \|  \|  \| \|  \| \|  \| \|  \|  \|  \|  \| **Table S3. Probit regression of treatment option on patient characteristics and therapies** \| \| \| \| \| \| \| \| \| \| --- \| --- \| --- \| --- \| --- \| --- \| --- \| --- \| --- \| \| **Treatment** \| \| **Coef** \| **Std. Err. (robust)** \| **z** \| **P>\|z\|** \| \| **95% CI** \| \| \| **Age** \| \| -.0077 \| .0151 \| -0.51 \| .609 \| \| -.0373 \| .0219 \| \| **Lymph node metastasis** \| \| -.6283 \| .4230 \| -1.49 \| .137 \| \| -1.4574 \| .2008 \| \| **Radiotherapy** \| \| .8746 \| .7221 \| 1.21 \| .226 \| \| -.5408 \| 2.2899 \| \| **ER positivity** \| \| .2740 \| .4156 \| 0.66 \| .510 \| \| -.5406 \| 1.0885 \| \| **Size** \| \| .0196 \| .1313 \| 0.15 \| .881 \| \| -.2377 \| .2770 \| \| **Constant** \| \| -.4779 \| 1.1704 \| -0.41 \| .683 \| \| -2.7719 \| 1.8160 \| \|  \|  \| \|  \|  \|  \| No. of obs = 64  Wald chi2 (5) = 4.39  Prob > chi2 = 0.4942  Pseudo R2 = 0.0519 \| \| \| \|  \|  \| \|  \|  \|  \| \|  \|  \| \|  \|  \|  \| | | | | |
